# Twelve Months Post-treatment Results From the Norwegian Version of Improving Access to Psychological Therapies

**DOI:** 10.3389/fpsyg.2019.02303

**Published:** 2019-10-18

**Authors:** Solbjørg Makalani Myrtveit Sæther, Marit Knapstad, Nick Grey, Otto R. F. Smith

**Affiliations:** ^1^Department of Health Promotion, Norwegian Institute of Public Health, Bergen, Norway; ^2^Department of Clinical Psychology, University of Bergen, Bergen, Norway; ^3^Sussex Partnership NHS Foundation Trust, Sussex, United Kingdom; ^4^School of Psychology, University of Sussex, Sussex, United Kingdom

**Keywords:** prompt mental health care, CBT, anxiety, depression, IAPT, real-life settings, long-term follow-up

## Abstract

**Objectives:**

Prompt Mental Health Care (PMHC) is the Norwegian version of the England’s Improving Access to Psychological Therapies (IAPT). Both programs have been associated with substantial symptom reductions from pre- to post-treatment. The present study extends these findings by investigating symptom levels at 12 months post-treatment, as well as treatment outcome in relation to low- vs. high-intensity treatment forms.

**Design and Outcome Measures:**

A prospective cohort design was used. All participants (*n* = 1530) were asked to complete the Patient Health Questionnaire-9 (PHQ-9) and the Generalized Anxiety Disorder-7 questionnaire (GAD-7) at baseline, before each session during treatment, at final treatment, and at 12 months post-treatment. Cohen’s d was used as effect size measure. Sensitivity analyses were conducted to examine the impact of the high missing data rates at post-treatment (≈44%) and 12 months post-treatment (≈58%).

**Results:**

A large symptom reduction was seen from baseline to 12 months post-treatment for both PHQ (*d* = −0.98) and GAD (*d* = −0.94). Improvements observed at post-treatment were largely maintained at 12 months post-treatment (PHQ (Δ*d* = 0.10) and GAD (Δ*d* = 0.09). Recovery rates decreased only slightly from 49.5% at post-treatment to 45.0% at follow-up. Both low- and high-intensity treatment forms were associated with substantial and lasting symptoms reductions (−1.26 ≤ *d* ≤ −0.73). Sensitivity analyses did not substantially alter the main results.

**Conclusion:**

The findings suggest long-lasting effects of the PMHC program and encourage the use of low-intensity treatment forms in PMHC like settings.

## Introduction

In 2006, “The depression report, A New Deal for Depression and Anxiety Disorders” by The Centre for Economic Performance’s Mental Health Policy Group gave compelling reasoning for upscaling the offer of evidence-based psychological therapy for individuals with anxiety and depression in England (Layard et al., 2006). The report takes us through how tremendous amounts of people experience clinically significant levels of anxiety and depression, how most are not treated – despite the existence of effective, evidence-based treatment, and finally, how such treatment would be cost-effective (Layard et al., 2006).

Aiming to improve access to evidence-based treatment for adults with anxiety disorders and depression in England, the program “Improving Access to Psychological Therapies” (IAPT) was initiated (Layard et al., 2006; Parry et al., 2011; Department of Health, 2012; Clark, 2018). IAPT provides treatment based on National Institute for Health and Care Excellence (NICE) guidelines, and utilizes both low intensity (e.g., guided self-help) and high intensity [e.g., individual cognitive behavioral therapy (CBT)] treatment forms (Clark et al., 2009; Chan and Adams, 2014; Clark, 2018). The program was first tested at two demonstrations sites, Doncaster and Newham (Clark et al., 2009; Parry et al., 2011), with promising results. About 55% of clients who had attended at least two sessions were classified as recovered when they left the services (Clark et al., 2009). Now the program has been established in virtually all Clinical Commissioning Groups in England, and the latest annual report showed that more than a million people are seen each year with an average recovery rate of 50.8% (NHS Digital, 2018).

Also in Norway, anxiety and depression are common in the adult population (Folkehelseinstituttet, 2018), important causes of functional impairment, sickness absence and disability pension (Gjesdal et al., 2008; Knudsen et al., 2010, 2012, 2013), and associated with high levels of disease burden (Global Burden of Disease Study 2013 Collaborators, 2015). Further, as in many other countries (Kohn et al., 2004; Thornicroft et al., 2017; Alonso et al., 2018), a large proportion of individuals experiencing anxiety and depression, are not being treated (Torvik et al., 2018). In order to address this, the Norwegian Ministry of Health and Care Services initiated an adapted version of the IAPT, “Prompt Mental Health Care” (PMHC, “Rask Psykisk Helsehjelp” in Norwegian), as a pilot project in 2012 (Helsedirektoratet, 2013; Smith et al., 2016). Like IAPT, the program is a free of charge, low threshold service aiming at short waiting times and requiring no referral from GP’s or other health care personnel (Helsedirektoratet, 2013; Smith et al., 2016).

The initial results from PMHC are promising, showing a large symptom reduction from baseline to final treatment (Smith et al., 2017; Knapstad et al., 2018). Effect sizes for both anxiety and depression were found to be around 1 (Smith et al., 2017; Knapstad et al., 2018), and recovery rates in line with the initial IAPT results from Doncaster and Newham (Parry et al., 2011; Smith et al., 2017). As these results are based on pilot cohort data with no comparison group, they should be interpreted with caution. However, previous randomized controlled trials with waitlist control groups have shown that spontaneous recovery rates tend to be high (>50%) for individuals with a relatively recent onset (<6 months) of anxiety and/or depression, but much lower (5–20%) for those with a longer duration (≥6 months) of clinically significant symptoms of anxiety and/or depression (Barton et al., 1975; Posternak and Miller, 2001; Clark et al., 2006). As about 80% of the PMHC clients reported symptoms to have lasted at least 6 months prior to treatment (Smith et al., 2016), the PMHC recovery rate was significantly higher than expected in similar (untreated) waitlist control groups (Knapstad et al., 2018).

Though PMHC is associated with initial improvement, symptom development beyond final treatment has not been reported. CBT is known to be a brief and effective treatment for depression and anxiety (Hofmann and Smits, 2008; Otte, 2011; Hofmann et al., 2012; Cuijpers et al., 2013), but it has been argued that short-term treatment might not always be enough to sustain improvement over time (Vittengl et al., 2007; Clark et al., 2018). In England, a follow-up survey was sent to a subgroup of clients in the two IAPT demonstration sites at least four months after treatment termination. Recovery rates were found to be 42% at follow-up compared to 57% at post-treatment in Newham (*n* = 60), and 50% at follow-up compared to 56% at post-treatment in Doncaster (*n* = 452) (Clark et al., 2009). This indicates that treatment gains at least in part are maintaned beyond final treatment. Examination of long-term effects is important in order to gain more knowledge about the potential treatment benefits for clients over time. This information can also aid the assessment of the programs’ cost-effectiveness.

In order to improve access to care through more efficient use of therapy resources, the use of low-intensity treatment forms has been advocated (Layard et al., 2006). Both stepped- and matched-care models have been proposed and are used for several mental health complaints (Newman, 2000; Scogin et al., 2003; Helsedirektoratet, 2013; Nordgreen et al., 2016). High intensity treatment forms are preferably reserved for individuals who do not benefit from, or are expected not to benefit from, low-intensity treatment (Bower and Gilbody, 2005). Stepped- and matched care models vary greatly with regards to type and number of interventions, and in criteria for stepping up (van Straten et al., 2015). In PMHC, information from the initial assessment and client’s wishes are used to determine type and intensity of care (Helsedirektoratet, 2013; Smith et al., 2016, 2017). This matched-care design means that the client does not always start with low intensity treatment (Helsedirektoratet, 2013). Treatment forms used in PHMC are all based on CBT and include individual treatment (defined as high intensity treatment), group-based psycho-education and guided self-help (defined as low-intensity treatments) (Helsedirektoratet, 2013; Smith et al., 2016, 2017).

Meta-analyses indicate that low-intensity treatment forms such as guided self-help based on CBT principles (Cuijpers et al., 2010, 2013), CBT delivered in groups (Huntley et al., 2012; Cuijpers et al., 2013; Barkowski et al., 2016; Burlingame et al., 2016) and computerized CBT (cCBT) (Andrews et al., 2010) are effective treatment forms for anxiety and depression disorders. Some studies even indicate that these treatment forms can give treatment outcomes comparable to that of individual treatment (Andrews et al., 2010; Cuijpers et al., 2010, 2013; Barkowski et al., 2016; Burlingame et al., 2016). Psycho-education for anxiety and depression seems to give promising results, although the evidence for group-based forms is scarce (Brown and Lewinsohn, 1984; Donker et al., 2009).

Although it seems that low-intensity treatment forms such as guided self-help and group-based psycho-education might be valuable alternatives to individual CBT, it should be noted that individuals included in studies investigating low intensity treatment forms often are recruited as volunteers (Spek et al., 2007; Andrews et al., 2010) and may as such not be representative for the typical PMHC client. It is therefore important to examine the impact of treatment forms on symptoms change in PMHC as well.

The present study aimed to investigate how symptoms of anxiety and depression developed from baseline to 12 months post-treatment among clients from the first 12 PMHC pilot sites. We also aimed to investigate whether symptom changes differed by treatment form (guided self-help, group-based psycho-education, individual CBT or a mixture of these treatment forms).

## Materials and Methods

### Pilot Sites and Procedure

The PMHC-pilots have been well described before (Helsedirektoratet, 2013; Smith et al., 2016, 2017; Knapstad et al., 2018). In short, the first 12 PMHC-pilot sites were established in 2012/2013, and were located across eastern, western and central Norway in both rural and urban areas. Demographic profiles and population size of catchment areas varied notably between pilot sites (Smith et al., 2016; Knapstad et al., 2018).

Independent of the catchment area, the PMHC teams had an average of four whole time equivalents. The teams were multidisciplinary, and included at least one psychologist with professional responsibility for the services. All therapists had at least 3 years of relevant higher education, and staff completed a 1-year training program in CBT (Knapstad et al., 2018). As mentioned above, clients could self-refer to PMHC, or be referred by their general practitioner (GP) or others health personnel. In this sample, 57% were referred by health personnel while 43% contacted PMHC themselves (Knapstad et al., 2018).

All clients participated in an initial assessment. They received information about PMHC and the therapist assessed the relevance and severity of mental health problems and decided whether PMHC could be the appropriate service (Knapstad et al., 2018). To be included, the patient had to be living in the pilot site community, be 18 years or older and have mental health needs related to anxiety and/or depression (no formal diagnosis was needed or provided) (Helsedirektoratet, 2013; Knapstad et al., 2018). Clients with a history or clear indications of psychosis, bipolar disorder, personality disorder, severe drug abuse, or suicide risk were generally referred to their GP or secondary health care services and not included in PMHC (Helsedirektoratet, 2013; Knapstad et al., 2018).

Participation in the study was based on opt-in. Clients who chose to participate signed informed consent and were asked to complete questionnaires before treatment (baseline), before each session during the treatment, at final treatment and at 12 months post-treatment (Knapstad et al., 2018). Participants who dropped out of treatment were still invited to complete the follow-up questionnaire. The median time from baseline to final treatment was 13.4 weeks (IQR: 7.0–22.1) and the median time from final treatment to follow-up was 55.0 weeks (IQR: 54.0–59.4 – throughout the manuscript referred to as “12 months post-treatment”). The Regional Ethics Committee for Western Norway approved the study (REK-vest no. 2014/597).

### Participants

In total, 2,512 clients started treatment at one of 12 the PMHC pilot sites between October 2014 and December 2016 (Knapstad et al., 2018). Of these, 1,530 (61%) signed informed consent and participated in the study (a previous article reported 1532 clients (Knapstad et al., 2018), but 2 of these cases turned out to be test clients). The participation rate varied across pilot sites, but was above 60% for 8 of the 12 sites (Knapstad et al., 2018). Of the 1,530 participants, 84.6% attended at least two treatment sessions. No information was available from those who received treatment, but did not participate.

As described previously (Knapstad et al., 2018), 75% of the PMHC clients were female. Most (54%) were between 26 and 44 years of age and 44% had completed higher education. More than 50% were married or cohabiting, 11% had immigrant background, 39% were working (full or part time) with no benefits, 35% had a job while receiving benefits, and 26% were not working, with or without benefits. Males, individuals over 67 years of age, individuals with lower education and individuals with immigrant background were underrepresented among participants compared to the population in the catchment areas (Knapstad et al., 2018). Upon inclusion, 84% of the clients reported their symptoms of anxiety and/or depression to have lasted 6 months or longer (Knapstad et al., 2018).

### Treatment Forms

The three main CBT-based treatment forms used in the PMHC pilots were guided self-help, where clients were guided in the use of self-help tools, such as literature and computer programs, group-based psycho-education, and face-to-face, individual CBT (Helsedirektoratet, 2013; Smith et al., 2016, 2017).

Information from the initial assessment, as well as client’s wishes, were used to determine treatment form (Helsedirektoratet, 2013; Smith et al., 2016, 2017). The therapists were asked to report treatment forms used at each meeting with the client (Smith et al., 2016).

If the therapist reported one of the above mentioned treatment forms to have been used in at least 80% of a client’s sessions, the client was placed in the corresponding group (guided self-help, group-based psycho-education, or individual CBT). The remaining clients were placed in the group “Mixed,” including clients who went from low- to high-intensity treatment or vice versa (Smith et al., 2016). To illustrate the latter, 36.3% of the clients in the mixed group received individual CBT as their first two sessions, whereas 33.1% received low-intensity treatment as their first two sessions.

Across pilot sites, individual CBT was the most used treatment form (Smith et al., 2016). Still, the included pilot sites differed in which treatment forms were most commonly used. Compared to the other pilot sites, two sites (Fosen and Molde) more often used guided self-help, and three pilot sites (Fjell, Notodden and Orkdal) more often used group-based psycho-education (Smith et al., 2016).

### Clinical Outcome Measures

As in IAPT, symptoms of depression and anxiety were measured using well-validated tools. Depressive symptoms were measured using the Patient Health Questionnaire -9 (PHQ-9) (Kroenke et al., 2001, 2010). Participants were asked how often during the last 2 weeks they had experienced nine common symptoms of depression, such as “little interest or pleasure in doing things” and “feeling tired or having little energy.” They reported the frequency on a scale from “not at all” (“0”) to “nearly every day” (“3”). The PHQ-9 has been shown to have good psychometric properties (Kroenke et al., 2001), and in our sample the Cronbach’s alpha for the instrument was 0.85 (Knapstad et al., 2018). A sum score was created, ranging from 0 to 27. Clients who had not answered all items, but at least 4, got their sum score based on the mean of the items they had responded to.

Anxiety was measured using the Generalized Anxiety Disorder-7 (GAD-7) (Spitzer et al., 2006; Kroenke et al., 2010). Participants were asked to rate how often during the last 2 weeks they had experienced 7 common symptoms of anxiety, such as “feeling nervous, anxious or on edge,” and “trouble relaxing.” The frequency was reported on the same scale as for PHQ-9, from “not at all” (“0”) to “nearly every day” (“3”). GAD has been found to have good reliability and validity for measuring generalized anxiety disorder (Spitzer et al., 2006), and to have satisfactory sensitivity and specificity for generalized anxiety as well as other anxiety disorders (Kroenke et al., 2007). In our sample, the Cronbach’s alpha for the instrument was 0.87 (Knapstad et al., 2018). A sum score was created, ranging from 0 to 21. As for PHQ, clients who had answered at least four items got their sum score based on the mean of the items they had responded to.

Clinical caseness was defined as having a PHQ-score ≥ 10 and/or a GAD-score ≥ 8, in line with the definition used in IAPT (Clark et al., 2009).

### Background Variables

Clients reported their gender (male, female), age (18–24, 25–44, 45–67, > 67), education (primary school, high school, university/university college), job status (working, working and receiving benefits, not working with or without benefits), marital status (living alone, not living alone) and immigrant background (yes, no).

Clients were also asked to rate their physical activity (days per week), their alcohol consumption (two times a week or more, once a week or less), and smoking status (yes, no). They also reported whether they were using antidepressant medication (every day, not every day), sleep medication (every week, not every week) or anxiolytic medication (every week, not every week). The perceived cause of symptoms was also reported (amongst others: relationship problems, school/job related, and/or bullying).

### Missing Data

As described previously, missing data rates at baseline were generally low (Knapstad et al., 2018), and 72.5% of the clients completed at least one follow-up questionnaire while under treatment (not including the final treatment questionnaire). However, the proportion of missing data was significantly higher at final treatment and 12 months post-treatment. The proportion of missing data at baseline, final treatment and 12 months was 1.04%, 43.5 and 57.9%, respectively, for PHQ, and 1.04, 43.7, and 59.1%, respectively, for GAD.

Missing data for PHQ and GAD at final treatment was associated with the baseline variables age, education, reporting the cause of current symptoms to be relationship problems or bullying, PHQ and GAD at the *p* < 0.10 level in logistic regression analyses including one predictor variable at the time, in addition to pilot site. In subsequent multiple logistic regression analyses only age, education and reporting the cause of current symptoms to be bullying predicted missingness at the *p* < 0.05 level. The McKelvey and Zavoina *R*^2^ was equal to 0.15. These three variables were only weakly correlated with PHQ and GAD scores at final treatment (*r* < 0.4).

Missing data for PHQ and GAD at 12 months was associated with baseline variables age, gender, marital status, job status, education, immigrant background, use of anxiolytic medication, and reporting the cause of current symptoms to be relationship problems or bullying at the *p* < 0.1 level in logistic regression analyses including one predictor variable at the time, in addition to pilot site. In subsequent multiple logistic regression analyses, gender, age, education and reporting the cause of current symptoms to be relationship problems or bullying predicted missingness at the *p* < 0.05 level. The McKelvey and Zavoina *R*^2^ was equal to 0.07. These five variables were only weakly correlated with PHQ and GAD scores at 12 months (*r* < 0.4).

These results provided some evidence that the data were partly missing at random (MAR). It is nonetheless likely that, in these types of settings, part of the missing data is missing not at random (MNAR) (Enders, 2010). Non-response for some participants may be more likely because of the actual (but unobserved) PHQ/GAD scores at post-treatment, which is conceivable for both those who are not improving and those recovering. The bias introduced by MNAR can partly be eliminated by including strong correlates of variables with missing data (Enders, 2010). In the present study, there were relatively strong relationships between the observed PHQ and GAD scores at post-treatment and, respectively, baseline PHQ and GAD scores (*r* ≈ 0.44), and the last observed measurement of PHQ and GAD under treatment (*r* ≈ 0.73). There were also strong associations between PHQ and GAD scores at 12 months post-treatment and, respectively, PHQ and GAD scores at post-treatment (*r* ≈ 0.50), and the last observed measurement of PHQ and GAD under treatment (*r* ≈ 0.50).

Information on therapist reported treatment form was missing for 26.7% of the clients. About 46% of the variance in missing therapist data was explained at the therapist level. This may suggest that some therapists have been more conscientious in reporting the required data than others. Cases with missing therapist data were excluded from the analyses when treatment form was included as an independent variable.

### Missing Data and Reason for Treatment Termination

Reasons for treatment termination were based on information from the questionnaires that the therapists provided by the end of treatment for each client. In 24.4% (*n* = 373) of the cases, no such information was provided by the therapists. Completers (67.1%, *n* = 1027) were defined as clients for whom the therapeutic goal was achieved (49.3%, *n* = 754) *OR* clients who attended at least six sessions (50.2%, *n* = 768 – note that these two criteria were not mutually exclusive). A cut-off at six sessions was used as this was the median number of treatment sessions for the entire sample and as this reflects a reasonable number of sessions for a complete short-term CBT-treatment. Forty-two clients were categorized as completers based on having completed at least six questionnaires during treatment even though no therapists-reported information was available.

Clients who did not meet these criteria were categorized as dropouts (7.3%, *n* = 112) or as being referred to other services (3.9%, *n* = 60), depending on the information provided by the therapists. The remaining 21.6% (*n* = 331) could not be categorized by reason for treatment termination.

Table 1 shows that non-response was much higher for dropouts, referrals and clients with missing therapist data. The mechanisms for non-response were likely different across groups. Not responding due to higher PHQ (or GAD) scores may be more likely among dropouts and referrals. The observed change for PHQ in this group was also much lower than the observed change in the completer group. For the group with missing therapist data, missing client data may to a lesser degree depend on actual PHQ (or GAD) scores, but may be more strongly associated with the degree to which the therapist committed him- or herself to the study – as a large percentage of the variance in missing therapist scores was explained at the therapist level. The observed change for PHQ in this group was also more similar to the observed change in the completer group (see Table 1).

**TABLE 1:** Data completeness across measurement occasions by reason for treatment termination.

	**Baseline**	**Under treatment**	**Post-treatment**	**12m. post-treatment**	**ΔPHQ post-treatment^∗^**
	
	**%(n)**	**%(n)**	**%(n)**	**%(n)**	**M(SD)**
All (*n* = 1530)	99.1(1516)	72.2(1109)	56.5(865)	42.3(647)	−6.2(5.5)
By reason for tr. termination:					
Completers (*n* = 1027)	99.8(1025)	86.3(886)	75.9(779)	50.2(516)	−6.4(5.3)
Drop-outs/referrals (*n* = 172)	100(172)	51.7(89)	37.8(65)	27.9(48)	−4.1(5.8)
Not reported (*n* = 331)	96.4(319)	40.4(134)	6.3(21)	25.1(83)	−6.4(7.8)

*^∗^: change in PHQ from baseline to post-treatment. M: mean change. SD: standard deviation.*

### Statistical Analyses

Stata version 15 was used for all analyses, unless otherwise stated.

Linear mixed models based on the available data from all participants (*n* = 1530) were used to examine the change in symptoms of anxiety and depression across four measurement occasions: pre-treatment, the last observed measurement under treatment, post-treatment, and 12 months post-treatment. Occasion was treated as a categorical variable using 12 months post-treatment as the reference category. Occasion was modeled as a random intercept, whereas dependency within pilot site was modeled as a (categorical) fixed effect. Effect sizes were calculated by dividing the mean difference score by the standard deviation at pre-treatment (Cohen’s *d*).

Mixed model analyses use maximum likelihood estimation and can handle data that are missing at random (MAR). Although there are no conclusive tests to prove the assumption of MAR, it is generally considered a more realistic assumption as compared to missing completely at random (MCAR) and provides less biased estimates than more traditional methods such as last observation carried forward. For clinical caseness, the STATA module *gsem* was used to estimate the mixed model. The marginal probabilities derived from this model provided a lower bound to the recovery rate that is commonly used in IAPT reports (Clark et al., 2009; National Health Service, 2017). Recovery rate is defined as the proportion of the number of clients that move from being at caseness at baseline to not at caseness at treatment termination divided by the total number of clients at caseness at baseline. In contrast to this definition, the gsem model did include clients not at caseness at baseline, and the marginal probabilities should therefore be interpreted as lower bounds for the recovery rate. That is, if some of the participants who were not at caseness at baseline moved to caseness at follow-up, the IAPT recovery rate would be somewhat higher than reported in the present study (National Health Service, 2017).

Two sensitivity analyses were conducted, using more conservative missing data strategies for clients that terminated treatment due to drop-out or further referral and for clients for whom the reason for treatment termination was unknown due to missing therapist data. The first sensitivity analysis adopted the last-observation-carried-forward strategy in the drop-outs and referral group (*n* = 172). Intuitively, this may be a realistic approach as additional change after the last observation seems less likely without additional treatment. The second sensitivity analysis extended the last-observation-carried-forward method to the group with missing information on reason for treatment termination (*n* = 331). Although this may be too conservative for this group, for reasons explained in the previous section, the large percentage of missing data, in particular at final treatment, warrants this approach and results could be considered as a reasonable worst-case scenario. It should be noted that this type of sensitivity analysis is primarily concerned with between-analysis differences (difference between regular and sensitivity analysis with regard to change between, for example, baseline and 12 months post-treatment). Differences within each sensitivity analysis across occasions become smaller and are as such less informative with regard to the sustainability of changes beyond post-treatment.

For outcomes PHQ and GAD, the likelihood ratio test was used to test for the interaction between occasion (baseline, last observation under treatment, final treatment and 12 months post-treatment) and treatment form (self-help, group-based, individuals and mixed). Adjusted models were estimated to account for baseline differences across treatment forms, including the PHQ/GAD score at baseline. As maximum likelihood missing values (MLMV) estimation was not available for multilevel models in Stata, Mplus version 8 was used to estimate the adjusted models for which baseline PHQ/GAD was used as a between-level covariate instead of an outcome variable in the multilevel model formulation. In order to obtain the same number of observations as for the unadjusted models, all covariates (see also next paragraph) were brought into the model as dependent variables under the assumption of multivariate normality.

Potential confounders of the association between therapy form and symptom change were identified by running a series of generalized linear models with treatment form as outcome and pilot site plus one baseline variable at the time as predictors. The association with treatment form was tested for each baseline variable mentioned in the measures section. Clients receiving individual CBT were set as reference category. A baseline variable was included as covariate in the adjusted models when the association was statistically significant at the *p* < 0.05 level (Wald test). In addition, prominent predictors of change that were identified in previous work (Knapstad et al., 2018) were included in the adjusted analyses (job status, immigrant background, use of antidepressant medication, use of sleep medication, and reporting bullying as cause of problems).

## Results

### Treatment Outcome at 12 Months Post-treatment

**FIGURE 1:**
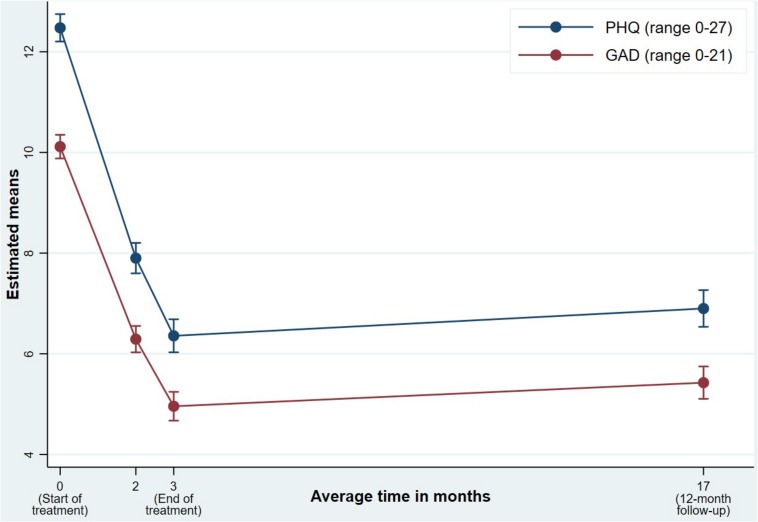
Course of anxiety and depression from baseline to 12 months post-treatment.

As shown in Table 2, PHQ and GAD levels at 12 months post-treatment continued to be much lower than the initial baseline levels (*d*_phq_ = -0.98 and *d*_gad_ = -0.94), despite a small increase in symptom levels from post-treatment to 12 months post-treatment (*d*_phq_ = 0.10 and *d*_gad_ = 0.09). This is visualized in Figure 1, in which the estimated means are plotted at each measurement occasion. The proportion of clients at caseness at 12 months post-treatment was 45% lower as compared to baseline. This should be considered as a lower-bound for the recovery rate used in IAPT. The proportion of clients at caseness increased with 4.5% from post-treatment to 12 months post-treatment.

**TABLE 2:** Change estimates using 12 months post-treatment as reference.

	**12m. post-treatment vs. Baseline**				**12m. post-treatment vs. Post-treatment**			
**Outcomes**	**Change**	**95% CI**	***p*-value**	**Effect size**	**Change**	**95% CI**	***p*-value**	**Effect size**
MAR-based analyses								
PHQ	–5.58	−5.93,−5.22	< 0.001	–0.98	0.54	0.15,0.93	0.006	0.10
GAD	–4.69	−5.01,−4.37	< 0.001	–0.94	0.47	0.12,0.82	0.008	0.09
Clinical caseness	–3.60	−3.98,−3.22	< 0.001	−45.0%^∗^	0.41	0.08,0.73	0.015	4.5%^†^
Sensitivity analyses 1								
PHQ	–5.13	−5.45,−4.81	< 0.001	–0.90	0.46	0.12,0.81	0.009	0.08
GAD	–4.32	−4.61,−4.04	< 0.001	–0.86	0.40	0.09,0.71	0.011	0.08
Clinical caseness	–3.76	−4.15,−3.38	< 0.001	−41.9%^∗^	0.30	−0.01,0.61	0.057	3.1%^†^
Sensitivity analyses 2								
PHQ	–4.38	−4.66,−4.11	< 0.001	–0.77	0.23	−0.05,0.51	0.110	0.04
GAD	–3.72	−3.96,−3.47	< 0.001	–0.74	0.19	−0.06,0.44	0.139	0.04
Clinical caseness	–4.08	−4.48,−3.68	< 0.001	−35.7%^∗^	0.14	−0.15,0.43	0.337	1.4%^†^

*MAR-based analyses: Linear mixed models assuming missing at random. Sensitivity analyses 1: LOCF strategy applied to drop-outs and those referred to other services (*n* = 172); Sensitivity analyses 2: LOCF strategy also applied to those for whom the reason for treatment termination was unknown (*n* = 331). ^∗^Proportion at caseness at 12 months post-treatment minus proportion at caseness at baseline. ^†^Proportion at caseness at 12 months post-treatment minus proportion at caseness at post-treatment.*

The results from the first sensitivity analyses using LOCF for drop-outs and referrals indicated somewhat lower effect sizes, but the changes from baseline to 12 months post-treatment could still be considered large (*d* > 0.8). The lower bound estimate for recovery rate was 41.9%. The second sensitivity analyses, using LOCF for clients with missing therapist data, indicated a further drop in effect size estimates. The change from baseline to 12 months post-treatment remained nonetheless in the close to large range (*d* ≈ 0.8). The lower bound estimate for recovery rate dropped to 35.7%.

### Treatment Forms

**FIGURE 2:**
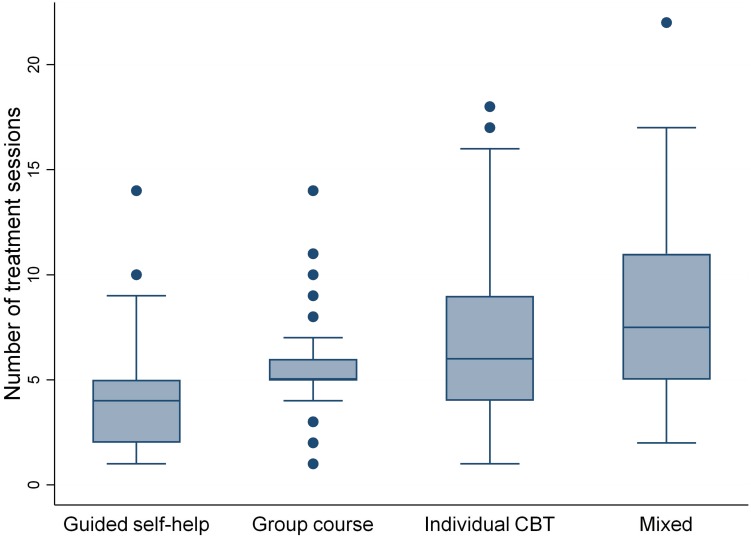
Box plot of number of sessions by treatment form.

Of the 1110 clients for whom the therapists reported information about treatment forms, 65.5% received primarily individual CBT, 15.8% received primarily group-based psychoeducation, and 7.8% received primarily guided self-help. The remaining 11.2% clients received a mixture of these three treatment forms. As displayed in Figure 2, the median number of treatment sessions was lowest for guided self-help (4, IQR = 2–5) and group-based psychoeducation (5, IQR = 5–6), and highest for individual CBT (6, IQR = 4–9) and mixed (7.5, IQR = 5–11).

Baseline differences across treatment forms were identified for age (χ^2^ (3) = 16.6, *p* < 0.001), bullying as cause of symptoms (χ^2^ (3) = 10.3, *p* = 0.02), and symptoms of anxiety (χ^2^ (3) = 13.6, *p* = 0.004). Clients receiving guided self-help or group-based psycho-education were on average older (≈40 vs. 37.0 years), and had lower average anxiety scores (≈9 vs. 10.2) as compared to those receiving individual CBT. Clients receiving group-based psycho-education more frequently reported bullying as cause of their symptoms as compared to clients receiving individual CBT (18.6% vs. 10.0%). Clients in the mixed group had higher average scores on anxiety compared to the individual CBT group (11.0 vs. 10.2).

### Treatment Outcome by Treatment Form

The likelihood-ratio test was statistically significant for both PHQ (χ^2^ (9) = 43.5, *p* < 0.001) and GAD (χ^2^ (9) = 55.8, *p* < 0.001), indicating different symptom trajectories across treatment forms. As shown in Table 3, the overall decrease in symptoms of depression and anxiety from baseline to 12 months post-treatment was large (*d* > 0.8) for all treatment forms. However, the estimated improvement was smaller for guided self-help (*d* = −0.88) and group-based psychoeducation (*d* = −0.87) as compared to individual CBT (*d* = −1.01). The estimated scores at 12 months post-treatment were not statistically different from each other, but this is likely due to lack of power as a consequence of substantial non-response at this occasion.

**TABLE 3:** Estimated scores at 12 months post-treatment and associated change compared to post-treatment and baseline by treatment form.

		**Estimated score at 12m. post-treatment**		**12m. post-treatment vs. Post-treatment**			**12m. post-treatment vs. Baseline**	
	**% (n)**	**Unadjusted (95% CI)**	**Adjusted (95% CI)**	**Unadjusted (95% CI)**	**Effect size**	**Adjusted (95% CI)**	**Unadjusted (95% CI)**	**Effect size**
**PHQ**								
Guided self-help	7.57(84)	7.30 (5.60, 9.00)	8.05 (6.56, 9.54)	1.97 (0.42, 3.52)	0.41	1.92 (0.45, 3.39)	−4.23 (−5.87, -2.58)^∗^	–0.88
Group-based psychoed.	15.77(175)	7.04 (5.89, 8.18)	7.27 (6.21, 8.33)	−0.27 (−1.35, 0.80)	–0.06	−0.07 (−1.12, 0.98)	−4.63 (−5.80, -3.45)^∗^	–0.87
Mixed	11.17(124)	5.80 (4.64, 6.95)	5.75 (4.71, 6.79)	−1.10 (−2.26, 0.07)^∗∗^	–0.20	−0.92 (−2.06, 0.21)^∗∗^	−7.08 (−8.45, -5.70)	–1.26
Individual CBT (Ref.)	65.50(727)	6.57 (6.03, 7.12)	6.64 (6.13, 7.15)	0.71 (0.20, 1.23)	0.12	0.71 (0.20, 1.22)	−5.97 (−6.55, -5.39)	–1.01
**GAD**								
Guided self-help	7.57(84)	5.63 (4.00, 7.30)	6.27 (4.71, 7.83)	2.06 (0.45, 3.67)	0.46	2.13 (0.46, 3.80)	−3.44 (−4.84, -2.05)^∗^	–0.76
Group-based psychoed.	15.77(175)	5.54 (4.65, 6.44)	5.80 (5.00, 6.61)	−0.31 (−1.24, 0.63)	–0.07	−0.24 (−1.15, 0.68)	−3.41 (−4.33, -2.49)^∗∗^	–0.73
Mixed	11.17(124)	4.89 (3.93, 5.86)	4.50 (3.62, 5.37)	−0.43 (−1.41, 0.54)	–0.08	−0.38 (−1.36, 0.60)	−6.04 (−7.10, -4.98)	–1.18
Individual CBT (Ref.)	65.50(727)	5.27 (4.79, 5.73)	5.34 (4.90, 5.79)	0.56 (0.10, 1.02)	0.12	0.58 (0.13, 1.03)	−5.01 (−5.53, -4.49)	–1.04

*^∗∗^Difference compared to individual CBT is significant at the *p* < 0.01 level. ^∗^Difference compared to individual CBT is significant at the *p* < 0.05 level. Variables included in the adjusted analyses: age, bullying as cause of problems, antidepressant medication, sleep medication, immigrant background, job status, and baseline PHQ/GAD (all grand mean centered). Effect size = unadjusted estimate/observed SD within treatment group.*

Guided self-help was associated with a clinically significant increase in symptoms of depression and anxiety from post-treatment to 12 months post-treatment (*d*_phq_ = 0.41, *d*_gad_ = 0.46), although this deterioration was not significantly different from the change between these two occasions for individual CBT (*d*_phq_ = 0.12, *d*_gad_ = 0.12). Again, this may be due to lack of power. Symptom levels were stable from post-treatment to 12 months post-treatment for the psycho-education group, whereas the mixed group improved somewhat further on symptoms of depression (*d* = −0.20). For depressive symptoms, this change was significantly different from the estimated change in the individual CBT group.

Adjusting for baseline differences across treatment forms and predictors of change did not alter the results substantially (Table 3).

## Discussion

Our findings indicate that improvements observed at post-treatment were largely maintained at 12 months post-treatment, despite a small, but statistically significant, increase in symptom levels. This is illustrated by estimated (lower bound) recovery rates being 49.5% at post-treatment and 45% at 12 months post-treatment. These findings are in line with a sub-group investigation from IAPT indicating that symptom improvement lasts beyond final treatment (Clark et al., 2009).

The sensitivity analyses also pointed to large and maintained decreases in symptoms of anxiety and depression at 12 months post-treatment. Given the level of missing data in the present study, carrying out sensitivity analyses is important. However, finding reasonable assumptions for these analyses is a challenging task, and can dramatically affect results (Enders, 2010). This seems especially true when there are high rates of missingness. In the present study, LOCF strategies were applied to clients who did not complete treatment and clients with unknown reason for treatment termination. The latter was used as a reasonable worst case scenario, and affected about one third of the sample. Also under these more conservative assumptions, the estimated change from baseline at 12 months post-treatment remained large. Although these sensitive analyses are useful in order to get an idea of the potential bandwidth of the estimates in the presence of MNAR, it’s important to keep in mind that maximum likelihood estimation, which we used for the primary analyses, is generally considered one of the preferred methods to analyze data with missing values, also in the presence of MNAR data (Siddiqui et al., 2009; Crameri et al., 2015).

Previous research has shown that low-intensity treatment forms can be effective for anxiety and depression (McDermut et al., 2001; Andrews et al., 2010; Cuijpers et al., 2010; Huntley et al., 2012; Cuijpers et al., 2013; Barkowski et al., 2016; Burlingame et al., 2016), though the evidence for *group-based* psycho-education is scarce (Brown and Lewinsohn, 1984; Donker et al., 2009). Our results showed that all treatment forms (guided self-help, group-based psycho-education, individual CBT and a mix of treatment forms) were associated with a large symptom reduction from baseline to 12 months post-treatment, although the low-intensity treatments were associated with a somewhat smaller reduction in symptom levels. Moreover, changes in symptoms from post-treatment to 12 months post-treatment were generally small across treatment forms. A notable exception was guided self-help, which was associated with a clinically significant increase in symptoms from post-treatment to 12 months post-treatment. By design, it’s difficult to determine whether the latter change is related to the actual treatment or to other client and/or therapist characteristics. Future studies should examine this potential problem in more detail. Overall, our results were in line with findings from IAPT which also found that both low and high intensity treatments were associated with a large reduction in symptoms from pre- to post-treatment (Chan and Adams, 2014). The current study extends these findings across treatment forms to results at long-term follow-up.

Neither in IAPT or in the current evaluation of PMHC, clients were randomized to treatment forms. In PMHC, client’s wishes and site preferences were decisive for treatment allocation. Our findings should therefore be interpreted with caution. To be able to provide unbiased comparisons of the effects across treatment forms, an equivalence or a non-inferiority trial should be considered. Nonetheless, that these treatment forms are associated with substantial improvement when employed to willing clients is also valuable information. In addition, baseline differences across treatment forms were not statistically significant for the majority of background variables, and accounting for significant differences did not alter the results. The differences in changes across treatment forms can therefore not be attributed to the included background variables, which to a certain extent increases the validity of our results.

The long-term, large reduction in symptoms associated with low-intensity treatment calls for an increased use of these treatments in PMHC, also as this study confirmed that these treatment forms were in fact low-intensity in terms of number of sessions used. Although frequent use of low-intensity treatment forms was an initial goal of PMHC (Smith et al., 2016), individual CBT was by far the most used treatment form across pilot sites (Smith et al., 2016; Knapstad et al., 2018). As discussed earlier (Smith et al., 2016; Knapstad et al., 2018), this might be related to therapists being primarily trained for individual, face-to-face treatment. Therapists may as such have been inclined to use face-to-face treatment instead of low-intensity treatments. Therefore, allocation to treatment may not have been solely based on information from the initial assessment and client‘s preferences, but also on therapist’s preferences. Also, guided self-help programs and group course materials were not available when the PMHC pilots started up. Considerable time and resources were required for development of material at local pilot sites, delaying and sometimes hampering implementation and use of low-intensity treatments (Smith et al., 2016; Knapstad et al., 2018). Moreover, use of guided self-help was not experienced as low-intensive by some therapists (Smith et al., 2016). In the light of these limitations reported at the pilot sites, the symptom improvement associated with low-intensity treatments can be considered promising. In order to increase the use of low-intensity treatments, and as such enable up-scaling and improvement of the cost-effectiveness of PMHC, evidence-based programs for guided self-help and group courses should be made more readily available. Evidence for the effectiveness of internet and other electronic interventions is also growing (Andrews et al., 2010), and may also have great potential for PMHC.

### Strengths and Limitations

An important limitation of the study is related to drop-out and missing data. Missing data rates were low at baseline, but high at final treatment and 12 months post-treatment. At 12 months post-treatment there was above 50% missing for the outcome measures of interest. This may have introduced bias. A compensating strength of the study was the use of the state-of-the-art methods to deal with these missing data. High levels of missing data also reduced power. In particular the power to detect meaningful differences between treatment forms was probably affected by this.

Additionally, two sensitivity analyses were performed to examine the potential impact of MNAR. The more realistic one of these two analyses, in which missing data among those who dropped out or were referred to other services were imputed by means of LOCF, indicated only a small decrease in change score estimates. This strengthens the finding from the main analyses; PMHC is associated with substantial and long-term improvement in terms of symptoms of anxiety and depression.

High attrition rates, in combination with a relatively low participation rate, resulted in a low overall response rate at 12 months post-treatment of about 25% (0.41 ^∗^0.61 ≈0.25). This limits the generalizability of the current findings. Although data for non-participants were unavailable, some selection among participants seems reasonable to assume (e.g., more females, less severe symptoms at baseline), which may potentially have affected the outcome estimates presented in the current paper.

Our analyses are based on cohort data from the first 12 PMHC pilot sites, and no control group was included. Though spontaneous recovery may be less likely in the PMHC sample, because the majority of participants reported symptoms to have lasted for at least 6 months prior to treatment, the initiated randomized controlled trial of PMHC (ClinicalTrials.gov Identifier: NCT03238872) will be of great value in demonstrating more precisely the short and long-term effects attributable to PMHC.

Guided self-help has been a core element from the start in the English and Australian versions of IAPT. It’s therefore likely that the pre-post treatment results of guided self-help in these countries are based on samples that included a broader range of clients as compared to the Norwegian sample. As such, our results may not generalize to IAPT programs that have guided self-help as primary mode of treatment.

Strengths of this study included a relatively large sample size, enabling investigation of treatment outcomes 12 months after final treatment, and comparison of treatment outcomes across treatment forms. Also, clinical outcomes were investigated using well-validated instruments (Kroenke et al., 2001, 2010; Spitzer et al., 2006). Finally, the pragmatic focus provides an encouraging demonstration of how treatments developed in controlled settings can be effective in a routine health care system.

## Conclusion

This study shows that improvement in symptoms of anxiety and depression achieved from baseline to post-treatment in PMHC were not temporary only. Both overall and across treatment forms, the change in symptom load from PMHC entry to 12 months post-treatment was large. As such, both low- and high-intensive treatment forms seem to be viable alternatives to help people who suffer from symptoms of anxiety and depression, even though the changes associated with low-intensity treatment forms were somewhat smaller. In order to improve cost-effectiveness of PMHC, increased use of low-intensity treatment forms, such as guided self-help and group-based psychoeducation, should be considered in the further roll-out of the service. Continuous monitoring of effectiveness should also be considered.

## Data Availability Statement

The datasets analyzed during the current study are not publicly available due to ethical restrictions and personal data protection, but are available from the corresponding author on reasonable request.

## Ethics Statement

The study was approved by the Regional Ethics Committee for Western Norway (REK-vest no. 2014/597). All participants have signed an informed consent scheme.

## Author Contributions

SS, MK, and OS designed the study, collected, analyzed, and interpreted the data, and drafted the manuscript. NG commented on early drafts of the manuscript. All authors contributed in interpretation of the data, offered critical revisions of the draft, and read and approved the final manuscript.

## Conflict of Interest

The authors declare that the research was conducted in the absence of any commercial or financial relationships that could be construed as a potential conflict of interest.

## Abbreviations

CBT: cognitive behavioral therapy
CI: confidence interval
ES: effect size
FU: follow-up
GAD: generalized anxiety disorder scale
GP: general practitioner
IAPT: increased access to psychological therapy
ITT: intention to treat sample
LOCF: last observation carried forward
MAR: missing at random
MCAR: missing completely at random
MNAR: missing not at random
NICE: National Institute for Health and Care Excellence
PHQ: patient health questionnaire
PMHC: prompt mental health care.
